# Determination of the Absolute Configuration of Secondary Alcohols in a Compound Mixture via the Application of Competing Enantioselective Acylation Coupled with LC/MS Analysis

**DOI:** 10.3390/pharmaceutics16030364

**Published:** 2024-03-05

**Authors:** Bum Soo Lee, Hoon Kim, Jiwon Baek, Rhim Ryoo, Seoung Rak Lee, Ki Hyun Kim

**Affiliations:** 1 School of Pharmacy, Sungkyunkwan University, Suwon 16419, Republic of Korea; kosboybs@naver.com (B.S.L.); wisekh@skku.edu (H.K.); baekd5nie@gmail.com (J.B.); 2 Department of Biopharmaceutical Convergence, Sungkyunkwan University, Suwon 16419, Republic of Korea; 3 Special Forest Products Division, Forest Bioresources Department, National Institute of Forest Science, Suwon 16631, Republic of Korea; rryoo@korea.kr; 4 College of Pharmacy and Research Institute for Drug Development, Pusan National University, Busan 46241, Republic of Korea

**Keywords:** *Podostroma cornu-damae*, CEA reaction, HBTM, LC/MS, secondary alcohol

## Abstract

The determination of natural product stereochemistry plays a significant role in drug discovery and development. Understanding the stereochemistry of natural products is essential for predicting and optimizing their interactions with biological targets, which, in turn, influences their therapeutic efficacy, safety, and overall impact on living organisms. Here, we present the first application of competitive enantioselective acylation (CEA) reactions in conjunction with LC/MS analysis for determining the absolute configuration of secondary alcohols in natural products which were purified as a mixture. This approach utilizes the enantiomeric pair of HBTM (homobenzotetramisole) catalysts, demonstrating sufficient kinetic resolution for the acylation of secondary alcohols. The rapid reaction kinetics were quantitatively estimated with LC/MS analysis as the characterization technique for the enantioselective transformations. Our study has expanded the application of the CEA reaction coupled with LC/MS analysis to mixtures. Utilizing LC/MS analysis, the CEA reaction offers a sensitive and simple method for stereochemistry determination. Additionally, the application of the CEA reaction is cost/time-effective since only small quantities of substrates and a short reaction time are required for characterizing the absolute configuration of secondary alcohols in natural products compared to other conventional methods.

## 1. Introduction

Natural products, encompassing a diverse array of secondary compounds derived from living organisms, often exhibit complex stereochemical features. The stereochemistry of a compound refers to the spatial arrangement of its atoms or groups in three-dimensional space. The stereochemistry of a compound influences its interactions with biological receptors, enzymes, and other molecular targets [1,2,3,4,5]. For example, enzymes often catalyze reactions by binding to specific substrates in a precise orientation. If the substrate’s stereochemistry does not match the enzyme’s binding site, the reaction may not occur or may proceed at a much slower rate. Similarly, drugs or ligands designed to interact with exact biological receptors must have the correct stereochemistry to exert their intended effects. Different stereoisomers of a natural product exhibit distinct biological activities, pharmacological properties, and even varying degrees of toxicity [6,7,8,9,10,11,12]. Even subtle differences in stereochemistry can have profound effects on a compound’s biological activity. For instance, two molecules with the same chemical structure but different stereochemistry may exhibit distinct pharmacological properties. The study of stereochemistry in natural products involves the analysis of chiral centers, stereoisomerism, and geometric isomerism. Understanding the stereochemistry of natural products is essential for medicinal chemists, pharmacologists, and researchers involved in drug discovery, providing insights into the design and optimization of bioactive compounds for therapeutic purposes [6,7,8,9,10,11,12].

Techniques such as NMR and circular dichroism spectroscopy, X-ray crystallography, and chemical reactions are commonly employed to elucidate the absolute configurations of structurally complex natural products [13]. Among these chemical reactions, Marfey’s reaction proves particularly valuable for determining the absolute configuration of amino acids in peptides [14,15], providing sensitive and specific results that aid in the discrimination between amino acid enantiomers [14,15]. Furthermore, the Mosher reaction has found extensive application in natural product chemistry, especially in establishing the absolute configuration of chiral alcohols [16,17]. This method offers a dependable and well-established approach for conducting stereochemical analysis, especially in situations where alternative methods may present challenges or yield inconclusive results [16,17]. Recently, micro-electron diffraction (Micro-ED) was developed and used for the structural determination of nanoscale materials including small organic molecules, peptides, proteins, and inorganic crystals [18,19,20]. Micro-ED enables the determination of the three-dimensional structures of natural products at the nanoscale level, even when they are present in very small quantities or as microcrystals. This is particularly advantageous for natural products that are challenging to crystallize or have limited availability [20].

Recently, we developed a method known as competing enantioselective acylation (CEA) coupled with LC/MS analysis for the absolute configuration determination of secondary alcohols in natural products [21]. This innovative approach relies on differential reaction rates with enantioselective catalysts for optically enriched substrates. Notably, the CEA method requires only around 30 min and micromole quantities of natural products for stereochemistry determination [21]. Moreover, the CEA method proves effective in establishing the absolute configuration of a secondary alcohol in natural products characterized by presence of multiple reactive functional groups together with a single secondary hydroxyl group [21]. By employing the CEA method, we definitively characterized the absolute configurations of secondary alcohols in pantheric acids A and B, isolated from a poisonous mushroom, *Amanita pantherine* [22]. In addition, the absolute configuration of a secondary alcohol of (3*R*,4*R*,5*S*)-obscurolide B1, identified from the termite-associated *Streptomyces neopeptinius* BYF101, was established by the application of the CEA approach [23]. Our recently developed method offers a highly sensitive, straightforward, and cost/time-efficient approach, providing a practical and convenient analytical method for establishing the absolute configuration of a single secondary alcohol in natural products.

As part of our ongoing studies to explore structurally novel natural products with bioactivity from intriguing natural sources [24,25], we investigated mycotoxins in the MeOH extract of the fungus *Podostroma cornu-damae*. This highly toxic mushroom belongs to the Hypocreaceae family and, due to its significant potential risk, is listed with the National Institute of Biological Resources [26]. Consuming *P. cornu-damae* can lead to early symptoms such as vomiting, dehydration, and diarrhea [27], with more severe effects like anuria, hypotension, polyps, leukopenia, thrombocytopenia, and impaired consciousness occurring around three days later [27]. In a prior study, we identified and explored the cytotoxic effects of eight macrocyclic trichothecenes, including three new compounds, from the plate cultivation of *P. cornu-damae* [28]. In our continuing efforts to pursue new toxic fungal metabolites from this poisonous mushroom, we obtained an inseparable mixture of roridin L-2 (**1**), a known trichothecene derivative, and a new α-hydroxy amino acid derivative, podostomide (**2**), through intensive chromatographic purifications and LC/MS and HR/MS-based analysis, coupled with an in-house UV spectrum library. The chemical structures of compounds in the mixture were characterized through interpretation of ^1^H nuclear magnetic resonance (NMR) and two-dimensional (2D) NMR spectroscopic studies, comprising ^1^H-^1^H correlation spectroscopy (COSY), heteronuclear multiple bond correlation (HMBC), heteronuclear single quantum correlation (HSQC), and rotating-frame Overhauser effect spectroscopy (ROESY). Since compounds **1** and **2** were purified as a mixture, we employed a competing enantioselective acylation (CEA) reaction to determine the absolute configuration at the secondary alcohol of **2**. This study presents the determination of the absolute configuration of secondary alcohols in a compound mixture using the CEA reaction coupled with LC/MS analysis. The successful application of this method in a compound mixture demonstrates its potential to broaden the scope of absolute configuration determination for secondary alcohols, even in mixtures of various natural products.

## 2. Materials and Methods

### 2.1. General Experimental Procedures

The equipment and devices used in the analyses and experimental procedures are listed in Table S1.

### 2.2. Chemicals and Reagents

All reactions were conducted in 20 mL vials using the organic solvent dimethylformamide (DMF) with air at room temperature. DMF and HBTM (*S*- and *R*-homobenzotetramisole) were employed as organic catalysts, *N*,*N*-diisopropylethylamine served as a non-nucleophilic base in organic chemistry, and propionic anhydride acted as an esterifying agent in acylation reactions. These chemicals were procured from Sigma-Aldrich Company (St. Louis, MO, USA).

### 2.3. Fungus Material

In 2020, we collected fresh fruiting bodies of *P. cornu-damae* from a forest in Pocheon, Republic of Korea. Isolation of the mycelium from the tissue of these fruiting bodies followed. Subsequently, the amplified sequence of the Internal Transcribed Spacer (ITS) region was compared to sequences in the NCBI Gene Bank. Upon analyzing the highest score and homology, the ITS sequence was confirmed as a match to *P. cornu-damae*.

### 2.4. Extraction and Isolation

*P. cornu-damae* was cultured on 300 PDA plates (90.0 × 15.0 mm) for 30 days at 25 °C. Once the plate was completely covered with mold, the media were combined in one place, chopped, crushed, and extracted overnight by pouring 100% MeOH into the combined state and repeating the process three times. After filtration and vacuum evaporation, a 42.4 g MeOH extract was obtained and dissolved in 700 mL of distilled water. Solvent partitioning was facilitated with hexane, CH_2_Cl_2_, EtOAc, and *n*-butanol (700 mL each), resulting in distinct layers at 0.5, 1.5, 0.5, and 2.8 g, respectively. LC/MS analysis revealed unknown compounds in the CH_2_Cl_2_ fraction, prompting further investigation. The CH_2_Cl_2_ fraction (1.5 g) underwent reversed-phase C_18_ open-column chromatography (MeOH/H_2_O; 30–100% MeOH), yielding five sub-fractions (A–E) based on TLC analysis. Sub-fraction B (0.3 g) was separated by open-column chromatography using Sephadex LH-20 (100% MeOH), resulting in four sub-fractions (B1–B4) by TLC analysis. Sub-fraction B2 underwent normal-phase silica open-column chromatography (CH_2_Cl_2_/MeOH; 30:1 → 0:1 *v*/*v*), yielding four sub-fractions (B21–B24) by TLC analysis. Sub-fraction B21 (17.2 mg) underwent reversed-phase semi-preparative HPLC with a phenyl-hexyl column (250 × 10.0 mm, 5 μm) manufactured by Phenomenex, utilizing an isocratic solvent system of 65% MeOH at a flow rate of 2 mL/min. Under these conditions, the HPLC separation resulted in a single mixture with 21.0 min of retention time, obtained in a quantity of 0.6 mg.

### 2.5. Competing Enantioselective Acylation (CEA) Coupled with LC/MS Analysis

Synchronized reactions were performed utilizing *S*- and *R*-HBTM stock solutions in the mixture. The procedures and conditions were consistent for both reactions, conducted on a blend of molecules, which are included in the Supplementary Materials. A 5 μL aliquot of the sample (100 μL), obtained from the parallel reactions, was directly introduced onto the LC/MS system for the identification of the acylated derivatives. The mobile phase condition for LC/MS analysis is provided in the Supplementary Materials. The reaction rate catalyzed by *S*- and *R*-HBTM was calculated by measuring the peak areas of the acylated derivatives.

### 2.6. Computational NMR–Chemical Shift Calculations for DP4+ Analysis

In the first stage, conformational searches were carried out utilizing MacroModel software (version 2021–4) manufactured by Schrödinger LLC. These searches were conducted within the MMFF94 force field, employing a combined torsional/low-mode sampling approach. Performed in a gas-phase setting, the searches utilized a 20 kJ mol^−1^ energy window with a maximum of 10,000 iterations. The subsequent conformational optimization employed the Polak–Ribiere conjugate gradient (PRCG) algorithm, with a convergence threshold of 0.001 kJ (mol Å)^−1^ and a maximum of 10,000 iterations for the root-mean-square gradient. The objective of the optimization was to minimize the acquired conformers. For further analysis in this study, the chosen conformers were limited to those falling within a 2 kJ mol^−1^ range in the MMFF force field. Subsequently, these conformers underwent geometry optimization using TmoleX 4.3.2 software, employing density functional theory (DFT) settings of B3-LYP/6-31+G(d,p) [29].

DP4+ calculations for isomer **2a** (9 conformers), **2b** (10 conformers), **2c** (10 conformers), and **2d** (9 conformers) conformers were performed at the theoretical level and basis sets. The determination of chemical shifts for **2a**, **2b**, **2c**, and **2d** involved the computation of magnetic shielding tensors using the following equation:

*δ^x^*_calc_ = σ^o^ − *σ^x^* (1)

In this context, *δ^x^*_calc_ represents the calculated chemical shift for nucleus x, *σ^x^* denotes the Boltzmann-averaged shielding tensor (averaged over all significantly populated conformations), and σ^o^ stands for the shielding tensor of tetramethylsilane, computed at the same level of theory as utilized for *σ^x^*. To interpret the experimental NMR data and ascertain the isomeric composition, we conducted DP4+ analysis. This process entailed the experimental NMR data comparison with the calculated Gauge-Independent Atomic Orbital (GIAO) NMR chemical shifts and *δ^x^*_calc_ values for potential isomers. The DP4+ analysis was executed utilizing an Excel sheet [30,31,32,33].

### 2.7. In Vitro Cytotoxicity Test

To assess the mixture’s cytotoxicity against four cancer cell lines, we employed a sulforhodamine B (SRB) bioassay [34,35]. The utilized cell lines in this investigation included (1) skin melanoma, SK-MEL-2, (2) ovarian malignant ascites, SK-OV-3, (3) non-small cell lung carcinoma, A549, and (4) colon adenocarcinoma, HCT. The cell lines were placed in conventional 96-well flat-bottom microplates and cultured for 24 h at 37 °C in a humidified environment with 5% CO_2_. Following this, the adherent cells underwent treatment with progressively diluted isolates and were further incubated for 72 h. Upon exposure to the mixture, the culture medium was aspirated, and the cells were fixed with 10% cold trichloroacetic acid for 1 h at 4 °C. Subsequently, the cells underwent rinsing with tap water and were stained using 0.4% SRB dye, followed by a 30 min incubation at room temperature. After washing with a 1% acetic acid solution, the cells were solubilized using a 10 mM unbuffered Tris base solution in pH 10.5. The absorbance at 520 nm was then measured using a microtiter plate reader manufactured by Molecular Devices (Sunnyvale, CA, USA). Doxorubicin (purity ≥ 98%), purchased from Sigma (Cleveland, OH, USA) was used as a positive control [36,37,38]. The IC_50_ values to determine cancer cell growth were calculated as the average of results from three independent experiments.

## 3. Results and Discussion

### 3.1. Planar Structural Elucidation of Compound Mixture

**Figure 1:**
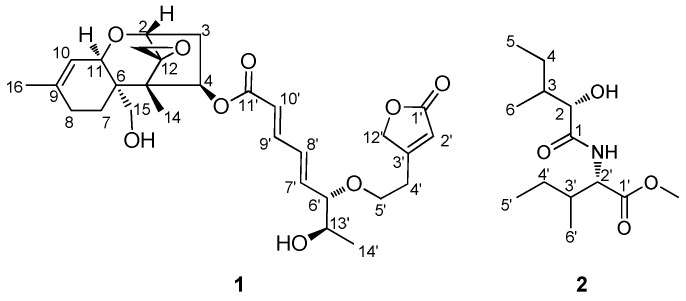
Chemical structures of compounds **1** and **2** in the mixture isolated from *P. cornu-damae*.

**Figure 2:**
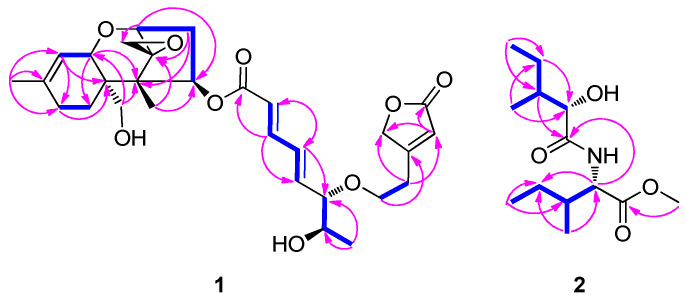
Key ^1^H-^1^H COSY (blue lines) and HMBC (pink arrows) correlation for compounds **1** and **2**.

An inseparable mixture of compounds **1** and **2** (Figure 1) was obtained as an amorphous powder, and their positive-ion mode HR-ESIMS data revealed two distinct protonated ion peaks at *m*/*z* 531.2599 [M + H]^+^ (calculated for C_29_H_39_O_9_, 531.2594) and 260.1859 [M + H]^+^ (calculated for C_13_H_26_NO_4_, 260.1862) (Figure S1), confirming that the two compounds were present as an inseparable mixture. From the HR-ESIMS data, the molecular formula of each compound was assigned to C_29_H_38_O_9_ and C_13_H_25_N_1_O_4_ for **1** and **2**, respectively. A thorough analysis of the ^1^H and 2D NMR experiments (^1^H-^1^H COSY, HMBC, HSQC, and ROESY) of the mixture allowed for the identification of **1** as roridin L-2 (Figure 2), previously reported from a study on fungal plant pathogen, *Myrothecium roridum* [39].

The ^1^H NMR spectrum (Table 1, Figure S2) of the mixture showed a typical chemical signal pattern for compound **1**, indicating a macrocyclic trichothecene-type compound [39]. The presence of an epoxide group [*δ*_H_ 2.83 (1H, d, *J* = 4.0 Hz, H-13a), 3.15 (1H, d, *J* = 4.0 Hz, H-13b)] and continuous two double bonds (H-7′, 8′, 9′, and 10′) existing in the 6.0–7.0 ppm range were confirmed, and it was also suggested that the structure of **1** was similar to that of the macrocyclic trichothecene-type compound. Key HMBC correlations of H-4′/C-2′, H-4′/C-12′, H-2′/C-1′, and H-2′/C-12′ of **1** led to the confirmation of the existence of 2-furanone moiety attached to C-4′. By detailed analysis of the ^1^H-^1^H COSY, HSQC, HMBC, and ROESY correlations (Figures S3–S6), the complete chemical structure of **1** was identified, as shown in Figure 1.

In addition to the NMR signals originating from **1**, we analyzed the remaining chemical signals of the mixture. The ^1^H NMR spectra of **2** revealed the presence of four methyls [*δ*_H_ 0.85 (3H, d, *J* = 7.0 Hz, H-6), 0.93 (3H, d, *J* = 7.0 Hz, H-6′), 0.93 (3H, t, *J* = 7.0 Hz, H-5′), and 0.97 (3H, t, *J* = 7.0 Hz, H-5)]; one methoxy [*δ*_H_ 3.75 (3H, s)]; two methylenes [*δ*_H_ 1.20 (1H, m, H-4′a), 1.35 (1H, m, H-4a), 1.46 (1H, m, H-4′b), and 1.47 (1H, m, H-4b)]; and four methines [*δ*_H_ 1.93 (1H, m, H-3), 1.93 (1H, m, H-3′), 4.16 (1H, m, H-2), and 4.63 (1H, dd, *J* = 9.0, 5.0 Hz, H-2′)]. The ^13^C NMR data, obtained from HSQC and HMBC analysis, showed the resonances of four methyls (*δ*_C_ 11.3, 11.7, 12.6, and 15.4), one methoxy (*δ*_C_ 51.5), two methylene (*δ*_C_ 25.3, and 26.1), four methine (*δ*_C_ 37.8, 37.8, 56.0, and 74.3), and two carbonyl carbons (*δ*_C_ 172.5 × 2). In the ^1^H-^1^H COSY data, vicinal proton correlations from H-2 to H_3_-6, along with HMBC correlations of H_3_-5/C-3, H_3_-6/C-2, H-3/C-1, and H-2/C-1, allowed for the identification of 2-hydroxy-3-methylpentanoic acid (Figure 2). Through detailed analysis of ^1^H-^1^H COSY and HMBC correlations, we confirmed connections from H-2′ to H_3_-6′, H_2_-4′/C-2′, H_3_-6′/C-2′, and H-3′/C-1′, which provide valuable insights into the isoleucine moiety. Additionally, an HMBC correlation of the methoxy group to C-1′ suggested that the methoxy group was attached to the carboxyl group at C-1′ in the isoleucine moiety. Finally, a key HMBC correlation of H-2′ to C-1 verified the linkage of the 2-hydroxy-3-methylpentanoic acid and isoleucine via an amide bond between C-1 and C-2′. The planar chemical structure of **2** was identified as depicted in Figure 1, trivially named as podostomide.

### 3.2. Determination of Absolute Configuration of Secondary Alcohols in Compound Mixture Using Competing Enantioselective Acylation Coupled with LC/MS

**Figure 3:**
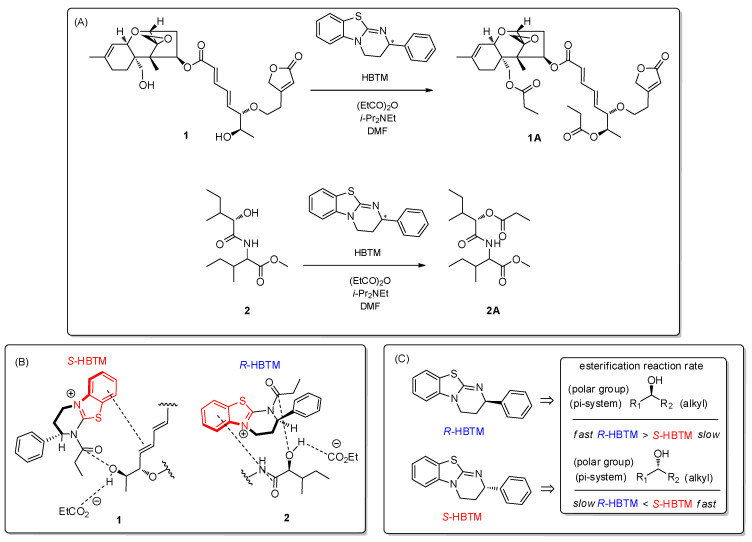
(**A**) CEA reaction to determine the absolute configuration of compounds **1** and **2**. The starred (*) carbons indicate chiral centers. (**B**) Proposed favorable transition state of compounds **1** and **2** in the reaction. (**C**) Key to predict the configuration of the secondary alcohols in the CEA reaction [21].

**Figure 4:**
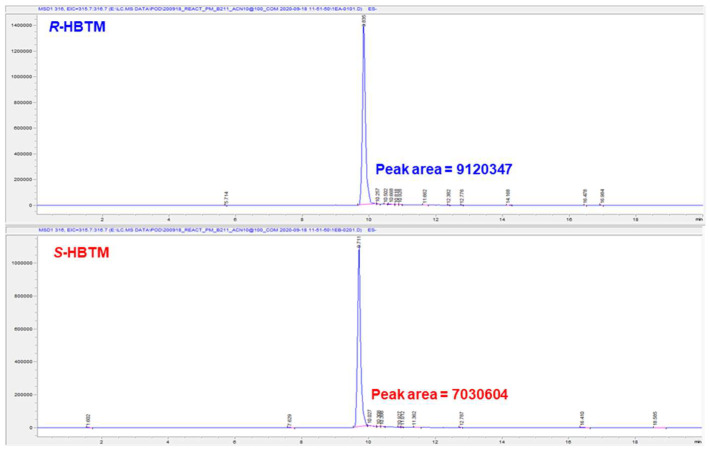
The extracted ion chromatograms (EIC, *m*/*z* 338 [M + Na]^+^) of acylated derivatives in *R*- and *S*-HBTM catalyzed acylation reaction of compound **2** at 20 min.

First, we considered the Mosher reaction, a stereoselective method commonly employed to assign the absolute configuration of natural products’ secondary alcohols. However, due to the fact that compounds **1** and **2** were obtained as a mixture in small amount (0.6 mg), the chiral derivatization reaction was deemed inappropriate for determining the absolute configuration at C-2 of **2**. Instead, we opted for the competing enantioselective acylation (CEA) reaction to determine the absolute configuration at C-2 of **2** in the mixture. The acylation of the secondary alcohol at C-2 of **2** was performed using an enantiomeric pair of homobenzotetramisole (HBTM) catalysts, providing sufficient kinetic resolution. Afterward, we compared the reaction rates of parallel reactions using LC/MS analysis as an indicator to prove enantioselective conversions. Through the utilization of LC/MS analysis, we estimated the reaction rates for acylation at C-2 in **2**, even though compounds **1** and **2** co-exist as a mixture. Two sets of the mixture (each 0.2 mg) containing *S*- and *R*-HBTM catalysts, respectively, were prepared, and the samples from each reaction were quantitatively analyzed by LC/MS to compare the reaction rate catalyzed by *S*- and *R*-HBTM. An acylated derivative **2A** ([M + Na]^+^ peak at *m*/*z* 338), esterified by propionic anhydride at C-2, was clearly detected (Figure 3). Additionally, the esterification reaction with *R*-HBTM appeared to occur more rapidly than that with *S*-HBTM through comparing the peak areas of the acylated derivatives in our LC/MS analysis (Figure S7). The extracted ion chromatograms (EIC, *m*/*z* 338 [M + Na]^+^) of acylated derivatives in reactions catalyzed by *R*- and *S*-HBTM of compound **2** at 20 min showed that the peak area (9,120,347) for an acylated derivative **2A** ([M + Na]+ peak at *m*/*z* 338) in the *R*-HBTM catalyzed acylation reaction was larger than that (7,030,604) in the *S*-HBTM catalyzed acylation reaction (Figure 4), indicating a faster esterification reaction with *R*-HBTM. The fast esterification with *R*-HBTM can be attributed to the hydroxy group at C-2 being positioned above the plane of **2** when the carbonyl group (π-system) is positioned to the left, and the alkyl group is situated to the right in the transition state (Figure 3B). Therefore, the absolute configuration of C-2 in **2** was assigned to be the *S*-form. Moreover, we observed that the esterification rate of **1** with *S*-HBTM was faster than that with *R*-HBTM. The rapid esterification with *S*-HBTM can be attributed to the secondary alcohol at C-13′ being positioned above the plane of **1** when the conjugated double bonds (π-system) are oriented to the right and the methyl group is positioned to the left in the transition state (Figure 3). This observation indicates that the absolute configuration of the secondary alcohol of **1** is in the *R*-form (Figure 3).

**Figure 5:**
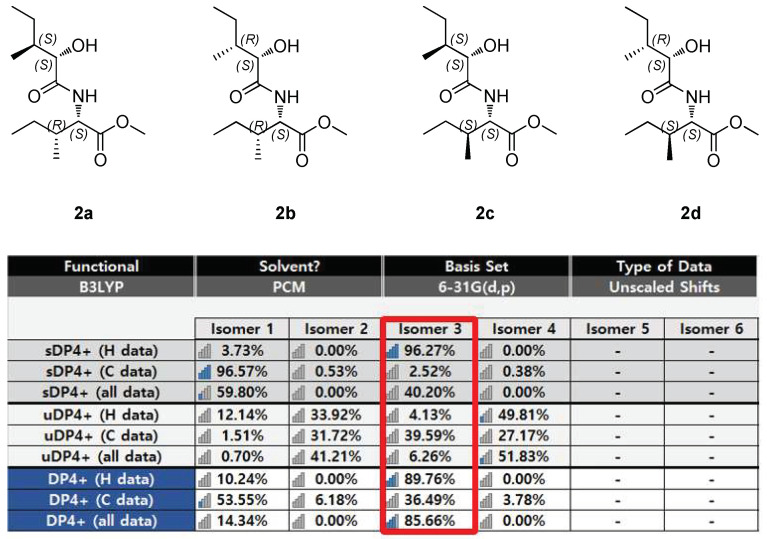
DP4+ analysis and probability scores for compound **2** with 4 isomers (**2a**, **2b**, **2c**, and **2d**). The red box indicates the isomer with the highest DP4+ probability score.

When we performed the large cultivation of *P. cornu-damae* on PDA plates, it was inferred that naturally occurring L-amino acids were supplied for the growth of the mushroom, suggesting that the isoleucine in the structure of **2** is in the L-form [40]. To elucidate the stereochemistry of the methyl at the C-3 and C-3′ positions of compound **2**, gauge-inclusive atomic orbital (GIAO) NMR chemical shift calculations were applied for the four possible isomers, combined with DP4+ probabilistic analysis. The computer-simulated ^1^H and ^13^C NMR chemical shifts of compounds **2a**, **2b**, **2c**, and **2d** were compared with the experimental NMR data of compound **2** by performing DP4+ analysis (Figure 5). The analysis revealed that compound **2c** (2*S*,3*S*,2′*S*,3′*S*) exhibited a high DP4+ probability score of 85.66% (Figure 5). It was confirmed that the assignment was highly reliable compared to other isomers. These results confirm the completely gross structure of compound **2**. Considering the stereochemistry assignment of the C-3 and C-3′ positions through computational calculations, it is noteworthy that 2,3,4,6-tetra-*O*-acetyl-β-D-glucopyranosyl isothiocyanate (GITC) derivatization for isoleucine and 2-hydroxy-3-methylpentanoic acid following the acid hydrolysis of **2**, coupled with LC/MS analysis of their GITC derivatives, can be employed to confirm the two chiral positions [41]. This approach may be particularly useful in future studies once a larger amount of sample can be obtained.

### 3.3. Evaluation of Cytotoxicity of the Mixture

In our earlier investigation, we assessed the in vitro cytotoxicity of isolated macrocyclic trichothecenes obtained from the MeOH extract of *P. cornu-damae* cultivation against human-derived breast cancer cell lines (MDA-MB-231, Bt549, MDA-MB-468, and HCC70) [28]. Certain macrocyclic trichothecenes demonstrated notable inhibitory effects on the breast cancer cell lines tested, displaying IC_50_ values (0.02–80 nM), which surpasses the potency of doxorubicin employed as a positive control. Given the origin of the mixture (compounds **1** and **2**) from the highly toxic mushroom *P. cornu-damae*, we proceeded to conduct additional in vitro cytotoxicity evaluations to explore the synergistic effect between compounds **1** and **2** in this study. The evaluation of cytotoxicity was conducted on four human-derived cancer cell lines (A549, SK-MEL-2, HCT15, and SK-OV-3) using the SRB bioassay [34,35], with doxorubicin employed as the positive control. However, we did not detect significant cytotoxicity (IC_50_ > 30.0).

## 4. Conclusions

In our ongoing efforts to discover toxic compounds from poisonous mushrooms, we have extended our investigation into the secondary metabolites of *P. cornu-damae*. Through successive purification steps and analysis of spectroscopic NMR and HR-ESIMS data, we isolated and structurally identified a mixture of two fungal compounds, including a novel α-hydroxy amino acid derivative named podostomide (**2**). To enhance our structural analysis, we firstly introduced the competing enantioselective acylation (CEA) reaction coupled with LC/MS analysis in a mixture. This pioneering application allowed us to determine the absolute configuration of a secondary alcohol in natural products obtained as a mixture. The success of this method reaffirms the practicality and simplicity of the CEA reaction, offering an efficient and time-effective approach for analyzing experimental results. In the application of CEA reaction, only small amounts of natural products (approximately 0.5 mg) and a relatively short reaction time (around 30 min) are needed to characterize the absolute configuration of secondary alcohols in natural products. Furthermore, through LC/MS analysis, the CEA reaction offers a sensitive and straightforward approach to determining stereochemistry. To our knowledge, there is currently no method utilizing sensitive LC/MS analysis for absolute configuration determination of natural products.

## Figures and Tables

**Table 1:** ^1^H NMR (850 MHz) and ^13^C NMR (212.5 MHz) data of compound **1** and **2** in CDCl_3_ ^a^.

	1			2	
Position	*δ*_C_	*δ*_H_ (*J* in Hz)	Position	*δ*_C_	*δ*_H_ (*J* in Hz)
2	78.8 CH	3.85, m	1	172.5 C	
3a	35.7 CH_2_	2.11, m	2	74.3 CH	4.16, m
3b		2.51, m	3	37.8 CH	1.93, m
4	75.5 CH	6.15, dd (8.5, 4.0)	4a	26.1 CH_2_	1.35, m
5	48.7 C		4b		1.47, m
6	43.9 C		5	11.7 CH_3_	0.97, t (7.0)
7a	21.1 CH_2_	1.55, m	6	12.6 CH_3_	0.85, d (7.0)
7b		1.98, m			
8	27.7 CH_2_	2.00, m	1′	172.5 C	
9	140.0 C		2′	56.0 CH	4.63, dd (9.0, 5.0)
10	118.7 CH	5.50, d (5.5)	3′	37.8 CH	1.93, m
11	66.6 CH	3.94, d (5.5)	4′a	25.3 CH_2_	1.20, m
12	65.6 C		4′b		1.46, m
13a	48.1 CH_2_	2.83, d (4.0)	5′	11.3 CH_3_	0.93, t (7.0)
13b		3.15, d (4.0)	6′	15.4 CH_3_	0.93, d (7.0)
14	6.3 CH_3_	0.84, s	OCH_3_	51.5 CH_3_	3.75, s
15a	62.8 CH_2_	3.84, m	NH		6.95, d (9.0)
15b		3.68, m			
16	23.1 CH_3_	1.73, s			
1′	173.7 C				
2′	116.6 CH	5.92, brs			
3′	166.9 C				
4′	29.0 CH_2_	2.72, dd (11.0, 5.5)			
5′a	66.0 CH_2_	3.56, m			
5′b		3.74, m			
6′	85.8 CH	3.58, m			
7′	138.5 CH	5.94, dd (15.5, 8.0)			
8′	132.3 CH	7.30, dd (15.5, 11.0)			
9′	143.5 CH	6.38, dd (15.5, 11.0)			
10′	122.4 CH	6.00, d (15.5)			
11′	167.4 C				
12′	72.9 CH_2_	4.79, s			
13′	69.4 CH	3.70, m			
14′	18.0 CH_3_	1.13, d (6.5)			

^a^ Coupling constants (Hz) are given in parentheses and ^13^C NMR data were assigned based on HSQC and HMBC experiments.

## Data Availability

Data are contained within the article and Supplementary Materials.

## Supplementary Materials

The following are available online at: https://www.mdpi.com/article/10.3390/pharmaceutics16030364/s1, Figure S1. The HR-ESIMS data of mixture of compounds 1 and 2. Figure S2. The 1H NMR spectrum of mixture of compounds 1 and 2 (CDCl3, 850 MHz. Figure S3. The 1H-1H COSY spectrum of mixture of compounds 1 and 2. Figure S4. The HSQC spectrum of mixture of compounds 1 and 2. Figure S5. The HMBC spectrum of mixture of compounds 1 and 2. Figure S6. The ROESY spectrum of mixture of compounds 1 and 2. Figure S7. The LC/MS data of acylated derivatives from CEA reaction at 20 min. Table S1. Equipment used for analyses.
