# Supplementary material for: Determination of the Absolute Configuration of Secondary Alcohols in a Compound Mixture via the Application of Competing Enantioselective Acylation Coupled with LC/MS Analysis
Source: Pharmaceutics. 2024 Mar 5;16(3):364. doi: 10.3390/pharmaceutics16030364 (PMC10974452; doi:10.3390/pharmaceutics16030364)
Supplement: Supplementary file 1 [file pharmaceutics-16-00364-s001.zip › pharmaceutics-2864858-supplementary.pdf]

Supplementary Materials

Determination of the Absolute Configuration of Secondary Alcohols in a Compound Mixture via the Application of Competing Enantioselective Acylation Coupled with LC/MS Analysis

Bum Soo Lee [1,†], Hoon Kim [1,2,†], Jiwon Baek [1], Rhim Ryoo [3], Seoung Rak Lee [4,*] and Ki Hyun Kim [1,*]

[1] School of Pharmacy, Sungkyunkwan University, Suwon 16419, Republic of Korea; kosboybs@naver.com (B.S.L.); wisekh@skku.edu (H.K.); baekd5nie@gmail.com (J.B.)
[2] Department of Biopharmaceutical Convergence, Sungkyunkwan University, Suwon 16419, Republic of Korea
[3] Special Forest Products Division, Forest Bioresources Department, National Institute of Forest Science, Suwon 16631, Republic of Korea; rryoo@korea.kr
[4] College of Pharmacy and Research Institute for Drug Development, Pusan National University, Busan 46241, Republic of Korea
* Correspondence: srlee17@pusan.ac.kr (S.R.L.); khkim83@skku.edu (K.H.K.); Tel.: +82-31-290-7700 (K.H.K.)
† These authors contributed equally to this study.

Supplementary Materials Contents:

Figure S1. The HR-ESIMS data of mixture of compounds **1** and **2**……………..….……………………………………………………………………….………………S3

Figure S2. The ^{1}H NMR spectrum of mixture of compounds **1** and **2** ($CDCl_3$, 850 MHz)........…..…..…………………………………………………………………….S4

Figure S3. The ^{1}H-^{1}H COSY spectrum of mixture of compounds **1** and **2**……………...……..…….……………………………………………………………………S5

Figure S4. The HSQC spectrum of mixture of compounds **1** and **2**...….……………… ….……………………………………………….…….………..…..………S6

Figure S5. The HMBC spectrum of mixture of compounds **1** and **2**……...……..…………………………….………………………………...………......….………S7

Figure S6. The ROESY spectrum of mixture of compounds **1** and **2**.........……………..……………......………………………………..…..…………..…..…….…..………S8

Figure S7. The LC/MS data of acylated derivatives from CEA reaction at 20 min….……………………………………………………………………………….S9

Materials and Methods ………..……………….S13

Figure S1. The HR-ESIMS data of mixture of compounds **1** and **2**

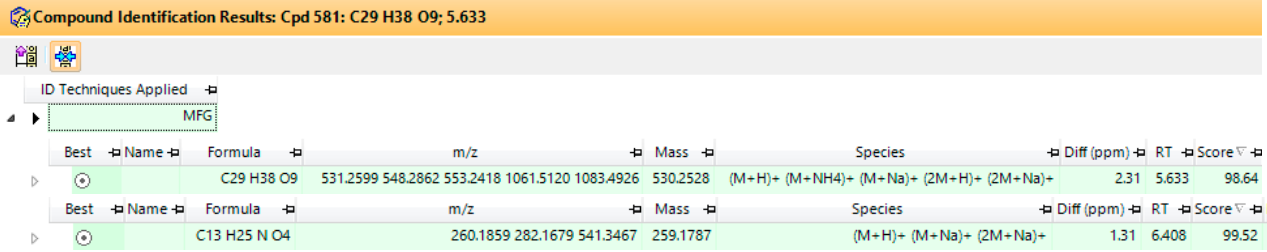

Compound Identification Results: Cpd 581: C29 H38 O9; 5.633

ID Techniques Applied: MFG

Best	Name	Formula	m/z	Mass	Species	Diff (ppm)	RT	Score
⊙		C29 H38 O9	531.2599 548.2862 553.2418 1061.5120 1083.4926	530.2528	(M+H)+ (M+NH4)+ (M+Na)+ (2M+H)+ (2M+Na)+	2.31	5.633	98.64

Best	Name	Formula	m/z	Mass	Species	Diff (ppm)	RT	Score
⊙		C13 H25 N O4	260.1859 282.1679 541.3467	259.1787	(M+H)+ (M+Na)+ (2M+Na)+	1.31	6.408	99.52

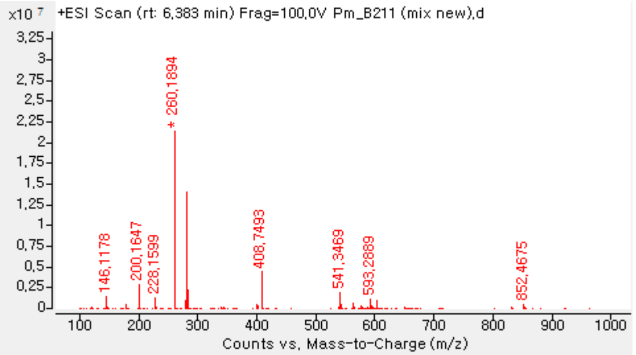

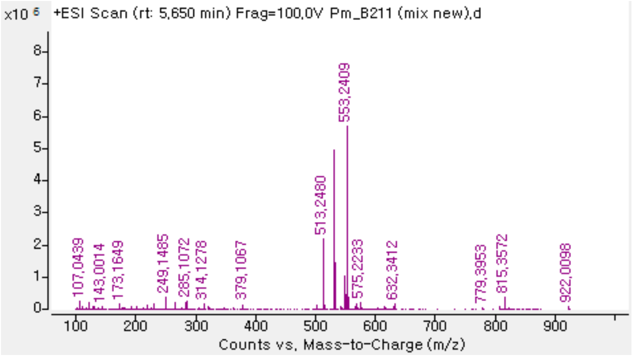

Figure S2. The ^{1}H NMR spectrum of mixture of compounds **1** and **2** ($CDCl_3$, 850 MHz)

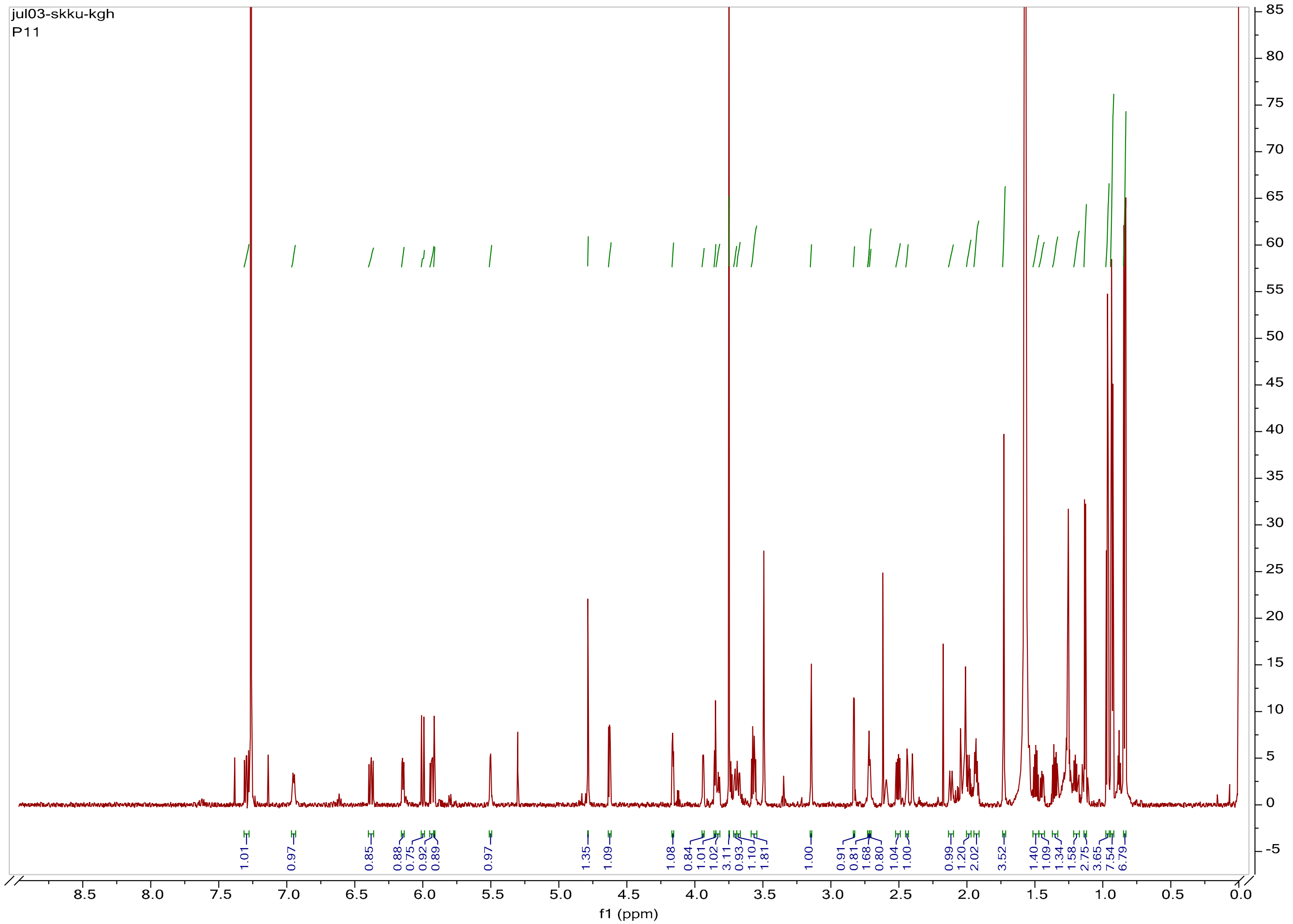

	Parameter	Value
1	Data File Name	C:/Users/생약학/Desktop/Total NMR/aug04-skku-kgh/10/fid
2	Title	aug04-skku-kgh
3	Comment	P-11
4	Origin	Bruker BioSpin GmbH
5	Owner	nmrsu
6	Site	
7	Spectrometer	spect
8	Author	
9	Solvent	CDCl3
10	Temperature	298.0
11	Pulse Sequence	zg30
12	Number of Scans	32
13	Receiver Gain	3
14	Relaxation Delay	1.0000
15	Pulse Width	8.9000
16	Acquisition Time	1.9268
17	Acquisition Date	2020-07-31T19:04:45
18	Modification Date	2020-07-31T19:04:44
19	Spectrometer Frequency	850.22
20	Spectral Width	17006.8
21	Lowest Frequency	-3419.9
22	Nucleus	1H
23	Acquired Size	32768
24	Spectral Size	65536

Figure S3. The ^{1}H-^{1}H COSY spectrum of mixture of compounds **1** and **2**

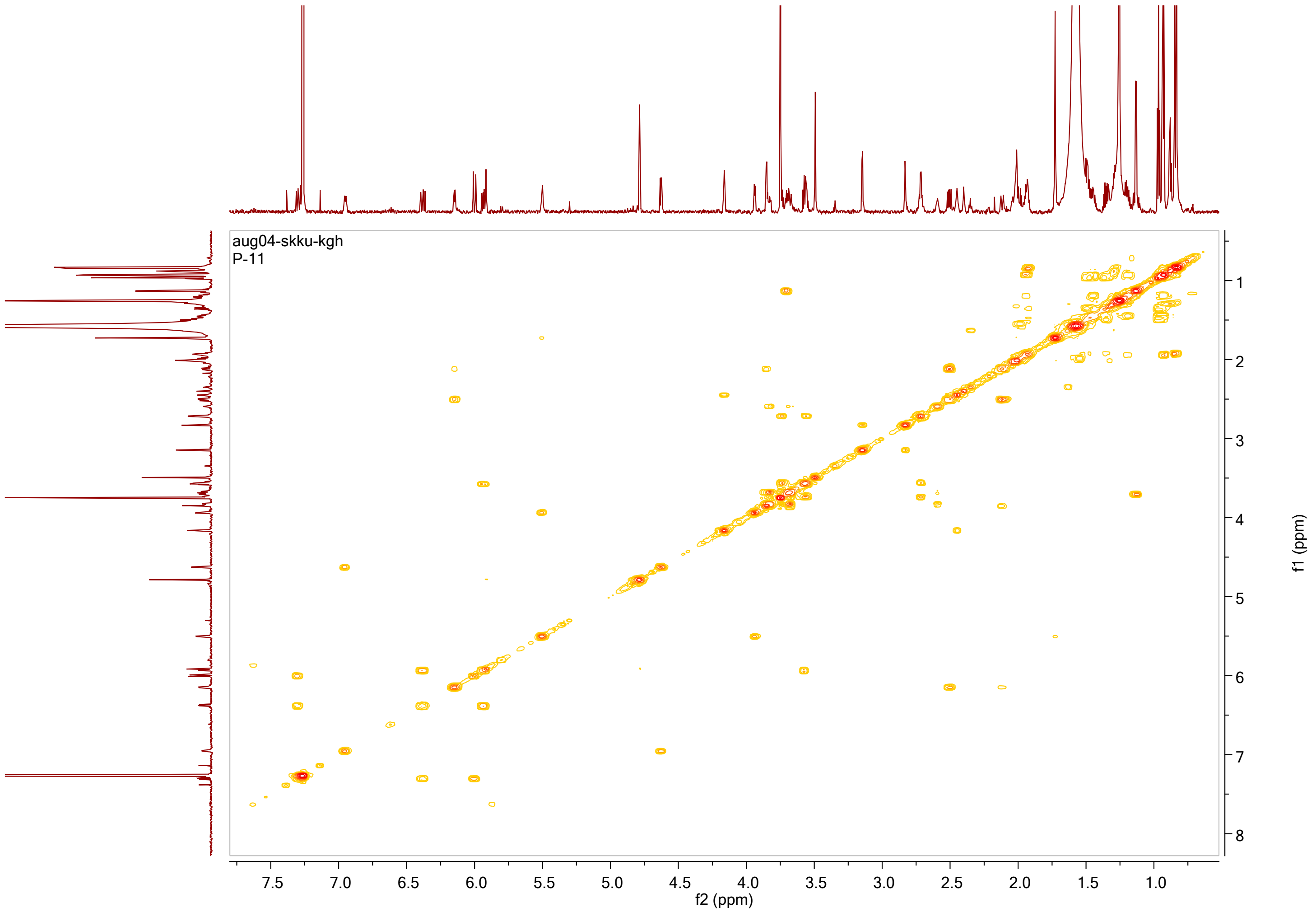

	Parameter	Value (f2, f1)
1	Data File Name	C:/Users/생약학/Desktop/Total NMR/aug04-skku-kgh/11/pdata/1/2rr
2	Title	aug04-skku-kgh
3	Comment	P-11
4	Origin	Bruker BioSpin GmbH
5	Owner	nmrsu
6	Site	
7	Spectrometer	spect
8	Author	
9	Solvent	CDCl3
10	Temperature	298.0
11	Pulse Sequence	cosygpppqf
12	Number of Scans	3
13	Receiver Gain	182
14	Relaxation Delay	2.0000
15	Pulse Width	7.5400
16	Acquisition Time	0.0754
17	Acquisition Date	2020-07-31T19:06:30
18	Modification Date	2020-07-31T19:33:36
19	Spectrometer Frequency	(850.22, 850.22)
20	Spectral Width	(13587.0, 13587.0)
21	Lowest Frequency	(-850.4, -850.4)
22	Nucleus	(1H, 1H)
23	Acquired Size	(1024, 256)
24	Spectral Size	(1024, 1024)

Figure S4. The HSQC spectrum of mixture of compounds **1** and **2**

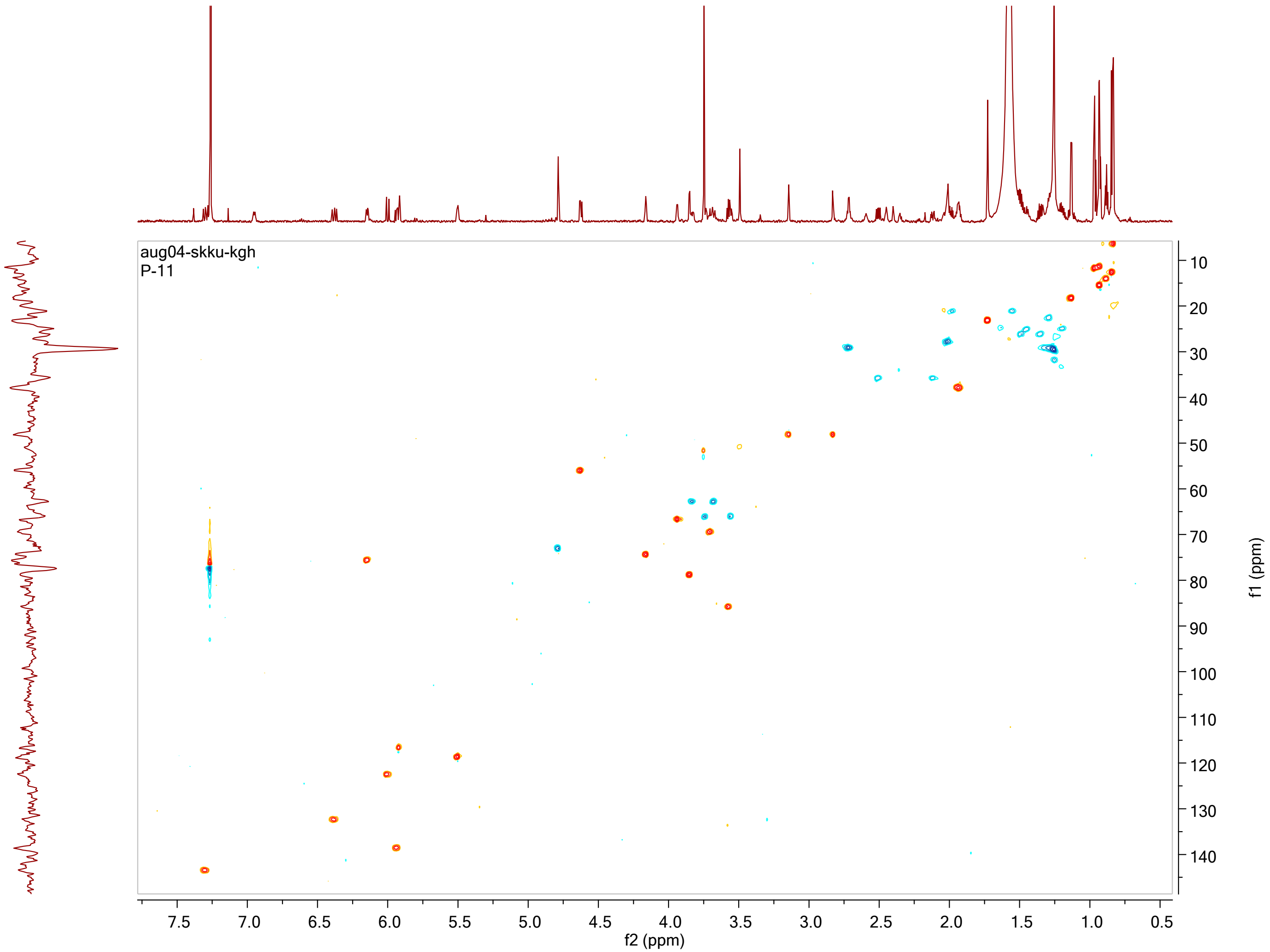

	Parameter	Value (f2, f1)
1	Data File Name	C:/Users/생약학/Desktop/Total NMR/aug04-skku-kgh/12/pdata/1/2rr
2	Title	aug04-skku-kgh
3	Comment	P-11
4	Origin	Bruker BioSpin GmbH
5	Owner	nmrsu
6	Site	
7	Spectrometer	spect
8	Author	
9	Solvent	CDCl3
10	Temperature	298.0
11	Pulse Sequence	hsqcedetgpsisp2.3
12	Number of Scans	4
13	Receiver Gain	182
14	Relaxation Delay	2.0000
15	Pulse Width	7.5300
16	Acquisition Time	0.0754
17	Acquisition Date	2020-07-31T19:36:55
18	Modification Date	2020-07-31T20:12:42
19	Spectrometer Frequency	(850.22, 213.79)
20	Spectral Width	(13587.0, 45045.0)
21	Lowest Frequency	(-847.0, -1143.7)
22	Nucleus	(1H, 13C)
23	Acquired Size	(1024, 256)
24	Spectral Size	(1024, 1024)

Figure S5. The HMBC spectrum of mixture of compounds **1** and **2**

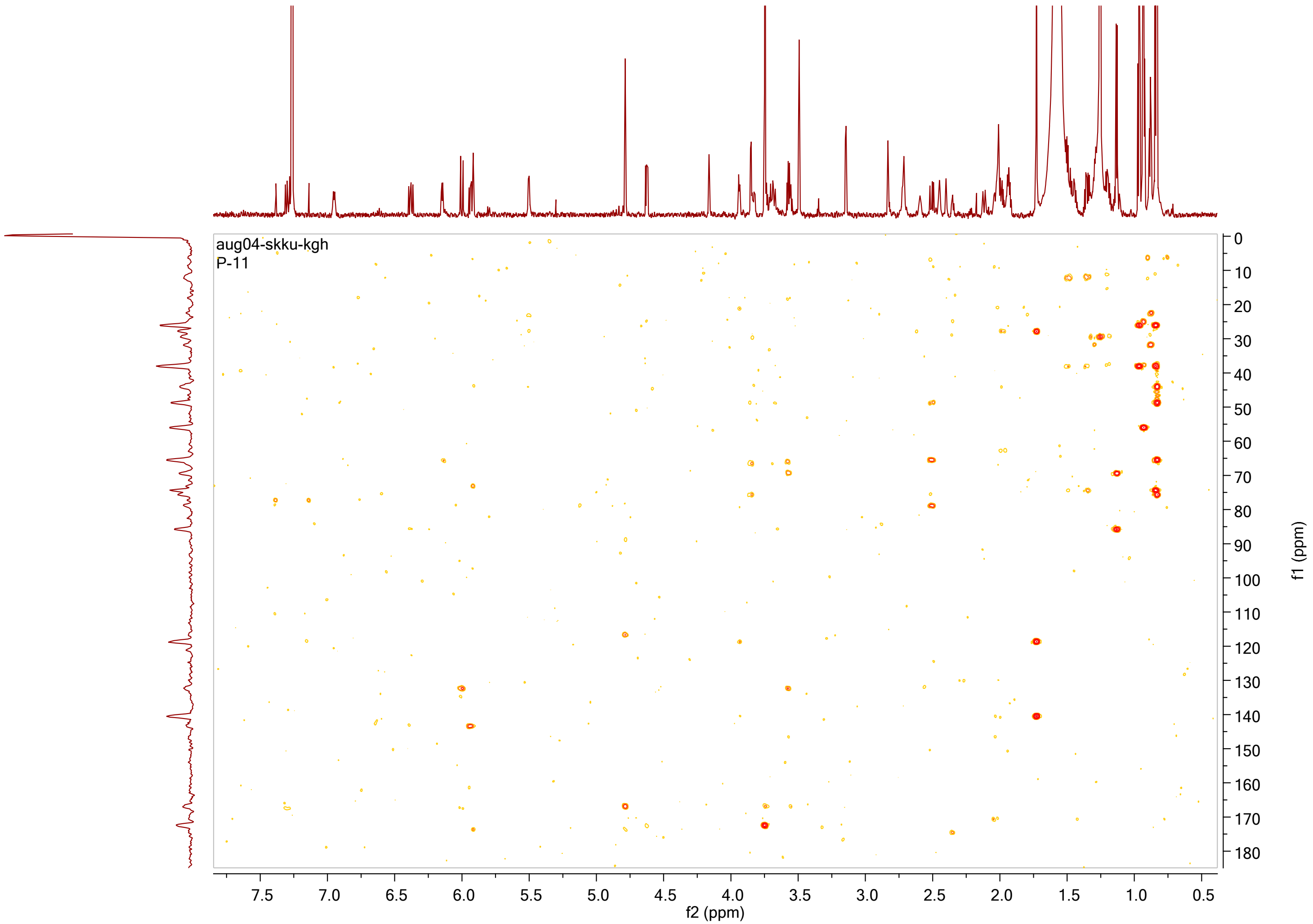

	Parameter	Value (f2, f1)
1	Data File Name	C:/Users/생약학/Desktop/Total NMR/aug04-skku-kgh/13/pdata/1/2rr
2	Title	aug04-skku-kgh
3	Comment	P-11
4	Origin	Bruker BioSpin GmbH
5	Owner	nmrsu
6	Site	
7	Spectrometer	spect
8	Author	
9	Solvent	CDCl3
10	Temperature	298.0
11	Pulse Sequence	hmbcetgpl3nd
12	Number of Scans	28
13	Receiver Gain	182
14	Relaxation Delay	2.0000
15	Pulse Width	7.5400
16	Acquisition Time	0.0754
17	Acquisition Date	2020-07-31T20:15:46
18	Modification Date	2020-08-01T00:32:28
19	Spectrometer Frequency	(850.22, 213.79)
20	Spectral Width	(13587.0, 51546.4)
21	Lowest Frequency	(-855.5, -2256.5)
22	Nucleus	(1H, 13C)
23	Acquired Size	(1024, 256)
24	Spectral Size	(2048, 1024)

Figure S6. The ROESY spectrum of mixture of compounds **1** and **2**

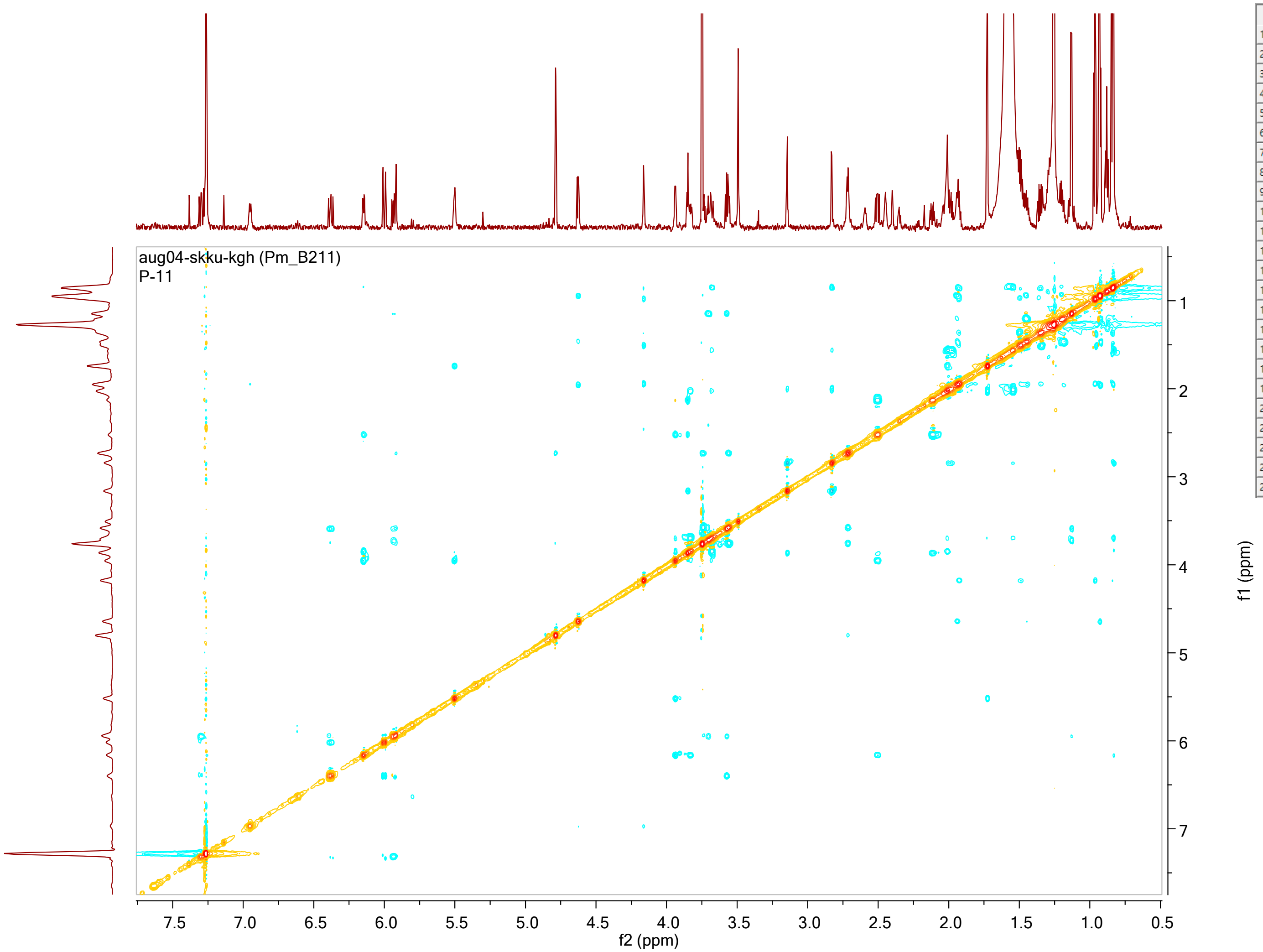

	Parameter	Value (f2, f1)
1	Data File Name	C:/Users/생약학/Desktop/Total NMR/aug04-skku-kgh (Pm_B211)/14/pdata/1/2rr
2	Title	aug04-skku-kgh (Pm_B211)
3	Comment	P-11
4	Origin	Bruker BioSpin GmbH
5	Owner	nmrsu
6	Site	
7	Spectrometer	spect
8	Author	
9	Solvent	CDCl3
10	Temperature	298.0
11	Pulse Sequence	roesyphpp.2
12	Number of Scans	8
13	Receiver Gain	182
14	Relaxation Delay	2.0000
15	Pulse Width	7.5400
16	Acquisition Time	0.1204
17	Acquisition Date	2020-08-03T13:35:45
18	Modification Date	2020-08-03T17:59:58
19	Spectrometer Frequency	(850.22, 850.22)
20	Spectral Width	(8503.4, 8503.4)
21	Lowest Frequency	(-865.3, -850.8)
22	Nucleus	(1H, 1H)
23	Acquired Size	(1024, 256)
24	Spectral Size	(1024, 1024)

Figure S7. The LC/MS data of acylated derivatives from CEA reaction

(a) A fully acylated derivative of compound **1** in *R*-HBTM catalyzed acylation reaction at 20 min

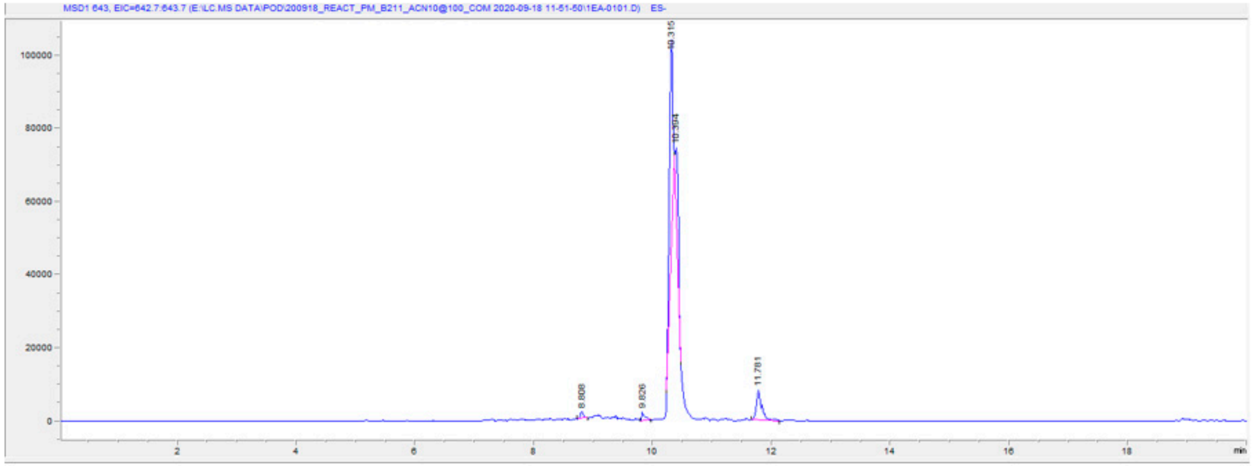

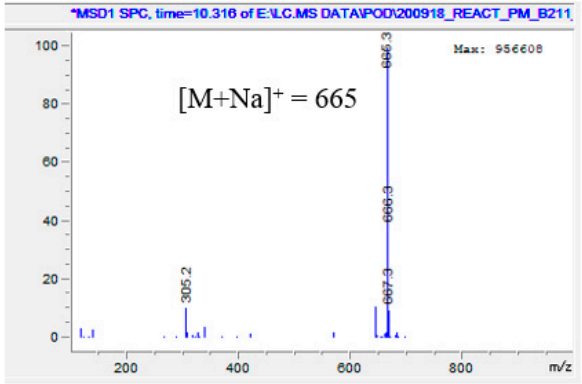

#	Time	Area	Height	Width	Area%	Symmetry
1	8.808	7819.4	1879.6	0.0684	2.120	0.888
2	9.826	10033.3	2433.5	0.0561	2.721	0.312
3	10.315	238416.3	59100.5	0.063	64.652	1.445
4	10.394	58755.4	14183	0.069	15.933	0.181
5	11.781	53743.1	8363.3	0.0909	14.574	0.512

(b) A fully acylated derivative of compound **1** in *S*-HBTM catalyzed acylation reaction at 20 min

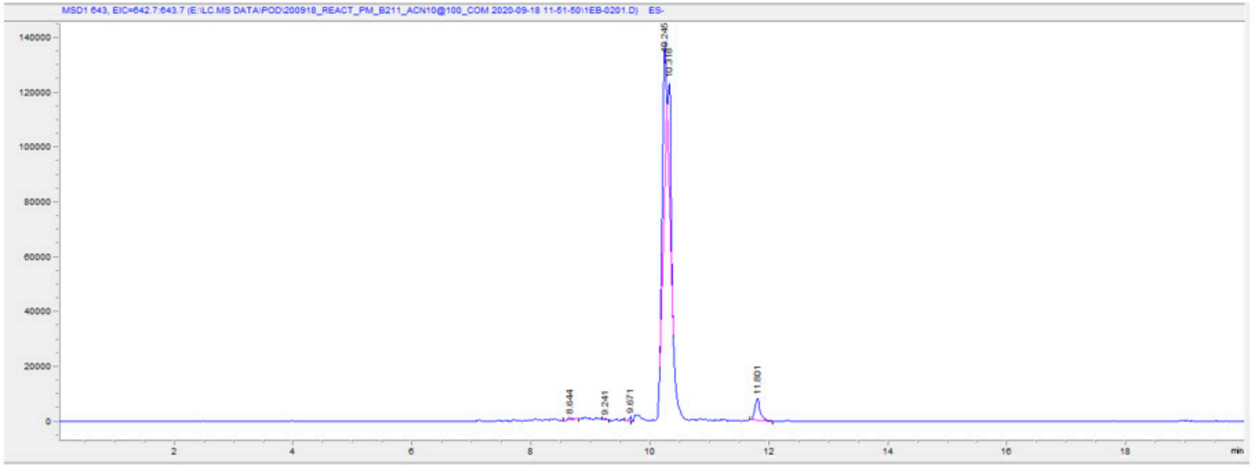

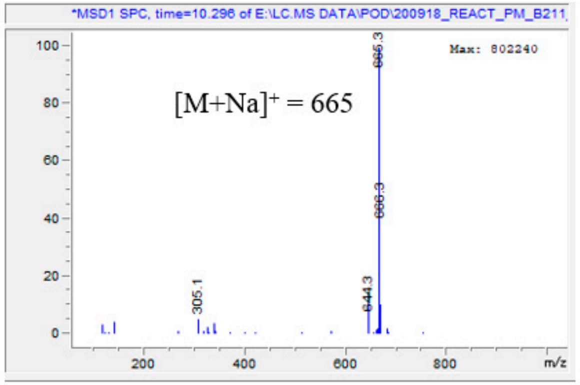

#	Time	Area	Height	Width	Area%	Symmetry
1	8.644	5570.8	1015.6	0.0799	1.290	0.559
2	9.241	1875.6	643.4	0.0499	0.434	0.697
3	9.671	3368.8	1928.3	0.0291	0.780	2.703
4	10.245	258469.3	58024.8	0.0719	59.838	2.095
5	10.318	113016.6	30973	0.0585	26.165	0.287
6	11.801	49645.2	8054.2	0.0918	11.493	0.724

(c) A fully acylated derivative of compound **2** in *R*-HBTM catalyzed acylation reaction at 20 min

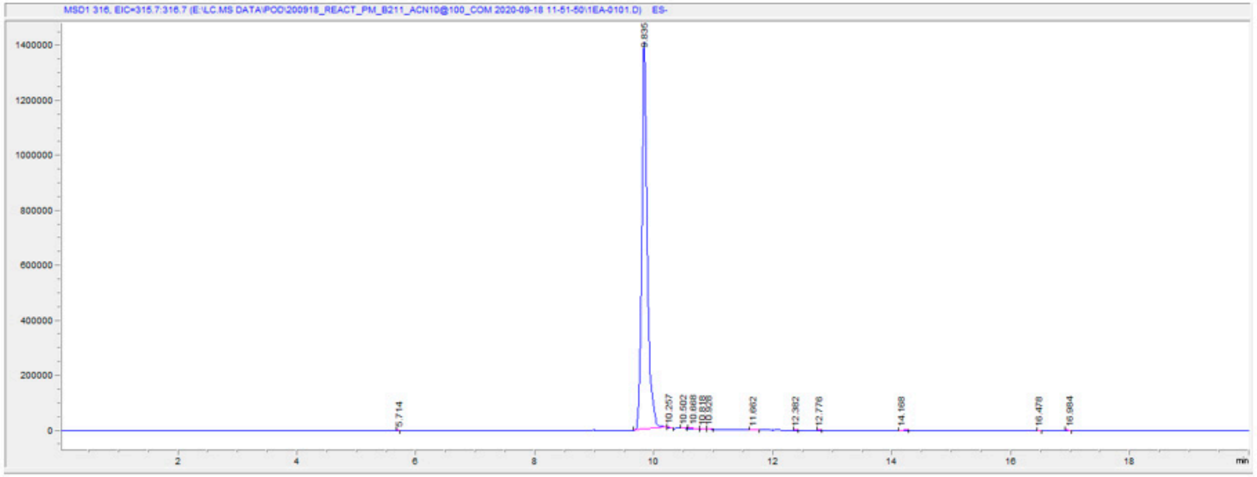

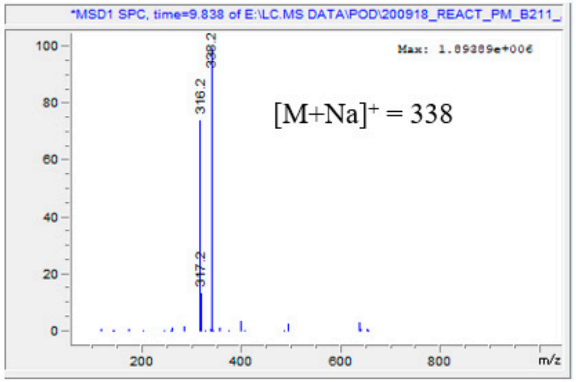

#	Time	Area	Height	Width	Area%	Symmetry
1	9.835	9120347	1415654.5	0.0951	100.000	0.765

(d) A fully acylated derivative of compound **2** in *S*-HBTM catalyzed acylation reaction at 20 min

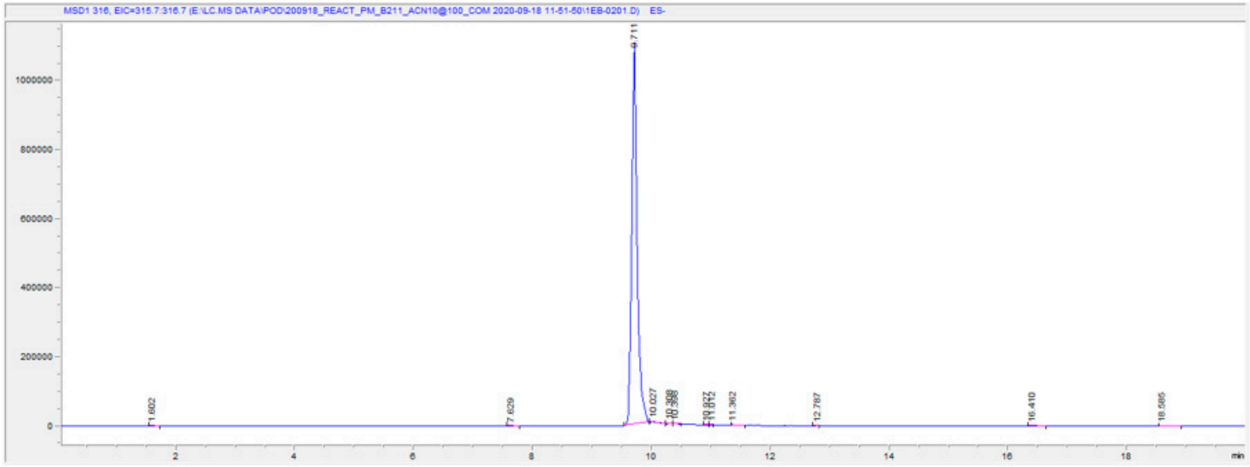

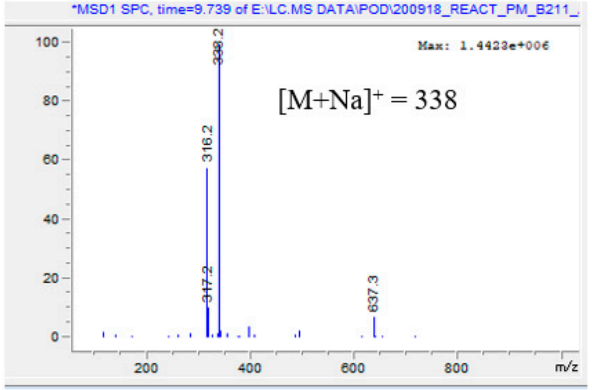

#	Time	Area	Height	Width	Area%	Symmetry
1	9.711	7030604	1106916.8	0.094	99.570	0.796
2	10.027	30359.4	3269.2	0.1203	0.430	0.179

Materials and Methods

Table S1. Equipment used for analyses.

Experimental procedure	Equipment
Optical rotations	Jasco P-2000 polarimeter manufactured by Jasco (Easton, MD, USA)
Ultraviolet (UV) spectra	Agilent 8453 UV-visible spectrophotometer manufactured by Agilent Technologies (Santa Clara, CA, USA)
Infrared (IR) spectra	Bruker IFS-66/S FT-IR spectrometer manufactured by Bruker (Karlsruhe, Germany)
Nuclear magnetic resonance (NMR) spectra	Bruker AVANCE III HD 850 NMR spectrometer manufactured by Bruker (Karlsruhe, Germany) with a 5 mm TCI CryoProbe operating at 850 MHz (^{1}H) and 212.5 MHz (^{13}C) with chemical shifts given in ppm (δ) for ^{1}H and ^{13}C NMR analyses.
HR-ESIMS	Agilent G6545B quadrupole time-of-flight mass spectrometer manufactured by Agilent Technologies with Agilent 1290 Infinity II high-performance liquid chromatography (HPLC) instrument using Agilent Eclipse Plus C18 column (2.1 × 50 mm, 1.8 µm) with flow rate of 0.3 mL/min
Open chromatography	Sephadex™ LH-20 manufactured by GE Healthcare Bio-Sciences AB (Uppsala, Sweden) with bead size of 25–100 µm
Semi-preparative HPLC	Shimadzu Prominence HPLC System with SPD-20A/20AV Series Prominence HPLC UV-Vis detectors manufactured by Shimadzu (Tokyo, Japan) and a Phenomenex Luna C18 column (250 × 10 mm, 5 µm) manufactured by Phenomenex (Torrance, CA, USA) wirh flow rate of 2 mL/min
LC/MS analysis	Agilent 1200 Series HPLC system equipped with a diode array detector and 6130 Series ESI mass spectrometer manufactured by Agilent Technologies using an analytical Kinetex C18 100 Å column (100 × 2.1 mm, 5 µm) manufactured by Phenomenex with flow rate of 0.3 mL/min.
Thin-layer chromatography (TLC)	Merck pre-coated silica gel F_{254} plates and RP-C_{18} F_{254s} plates manufactured by Merck; spots detection, under UV light or by heating after spraying with anisaldehyde-sulfuric acid.

Competing enantioselective acylation (CEA) coupled with LC/MS analysis

Synchronized reactions were performed utilizing *S*- and *R*-HBTM stock solutions in the mixture. The procedures and conditions were consistent for both reactions, conducted on a blend of molecules. The mixture, encompassing compounds **1** and **2** (0.4 mg), was distributed into two labeled 5 mL transparent capped vials at room temperature. Dimethylformamide (90 μL) was introduced as the organic solvent for the CEA reaction. Subsequently, stock solutions of both *S*- and *R*-HBTM (10 μL each) were added, followed by the sequential transfer of the *N*,*N*-diisopropylethylamine stock solution (2.9 μL). To initiate the CEA reaction, propionic anhydride (2.1 μL) was incorporated. Throughout the reactions, 2 μL aliquots from each reaction were obtained at various time intervals for LC/MS analysis and quenched with 98 μL of MeOH to attain a total volume of 100 μL. The reaction was halted by adding MeOH after 20 minutes.

A 5 μL aliquot of the sample (100 μL), obtained from parallel reactions at different time intervals, was directly introduced onto the LC/MS system with Phenomenex Luna C18 column (2.1 × 100 mm, 5 μm) at 0.3 mL/min flow rate. Full scans in positive- and negative-ion modes (scan range *m/z* 100−1000) were employed for the identification of the desired acylated derivatives. The mobile phase, consisting of 0.1% (v/v) formic acid in distilled water (A) or MeOH (B), was applied using a gradient solvent system: starting with 10% B and increasing to 100% B over 10 minutes, maintaining 100% B isocratically for the next 10 minutes, and finally returning to 10% B isocratically for 5 minutes to facilitate a post-run washing procedure for the column. The determination of the reaction rate catalyzed by both *S*- and *R*-HBTM involved measuring the peak areas of fully acylated derivatives.
